# Bioactive Compounds in Foods: New and Novel Sources, Characterization, Strategies, and Applications

**DOI:** 10.3390/foods14091617

**Published:** 2025-05-03

**Authors:** Ivana Generalić Mekinić, Vida Šimat

**Affiliations:** 1 Department of Food Technology and Biotechnology, Faculty of Chemistry and Technology, University of Split, R. Boškovića 35, 21000 Split, Croatia; 2 University Department of Marine Studies, University of Split, R. Boškovića 37, 21000 Split, Croatia; vida@unist.hr

The current global population has reached almost 8 billion, and it is predicted that by 2050, this number will rise to 9.6 billion, representing a serious challenge for the food industry in the future. According to the UN, the demand for food will increase by almost 70% by that time, accentuating problems such as malnutrition, food insecurity and food shortages in certain regions of the world, as well as the rise of lifestyle- and diet-related diseases and an increasingly pronounced economic imbalance [1,2,3]. Global agricultural production is also adapting to climate changes, unsustainable agricultural practices, environmental degradation, technological challenges, biodiversity loss, depletion and poor management of water resources, among other concerns [4,5]. All of the above exert severe pressure on global food production, which, in addition to the mentioned challenges, needs to adapt to current food trends and ensure that the safety and quality of food products are maintained [6]. Only through innovations in the food sector, such as new preservation methods, the development of healthier and functional products, processing steps that can enhance and enrich the nutritional profile of products, finding novel sources food, natural preservatives, and the development of clean label products is it possible to overcome these challenges [7].

Bioactive compounds are highly valuable non-nutrient components that exert physiological effects, which are often protective and beneficial for human health. These include diverse phytochemicals, volatile metabolites, and even micronutrients and microbe-derived compounds. Epidemiological evidence links diets rich in bioactives (for example, the Mediterranean diet) with lower incidence of cardiovascular, metabolic, and neurodegenerative diseases, as well as cancer [8,9,10]. Over the last five years (2020–2025), research has rapidly advanced with regard to identifying these compounds, understanding their biological activities, and developing technologies for their integration into food products. This editorial synthesizes recent findings on food bioactive compounds, their sources, characterization strategies, food industry applications, and implications for human health. Well-established sources of bioactive molecules include fruits, vegetables, grains, legumes, herbs, and fermented foods, which are rich in flavonoids, phenolic acids, carotenoids, glucosinolates, alkaloids, vitamins, and probiotics [10]. Volatile organic compounds in foods are typically associated with aroma and flavor, but many also possess bioactivity. Essential oils and flavor components from herbs, spices, fruits, and other botanicals contain terpenes, aldehydes, and phenolics that exhibit antioxidant, anti-inflammatory, and antimicrobial properties [11]. Traditional nutrients like vitamins, minerals, and peptides can also be considered bioactive when they perform regulatory functions or confer protection beyond basic nutrition. Also, beneficial microbes used in food fermentations—especially lactic acid bacteria (LAB) and certain *Bacillus* or yeast strains—produce a variety of metabolites with bioactive properties [12]. Across all these categories, bioactive compounds often serve functional roles in food systems in addition to their biological activities. Many phytochemicals are pigments (such as anthocyanins and carotenoids) that impart appealing colors, or antioxidants that delay lipid oxidation and spoilage, thereby naturally preserving foods. Volatiles contribute to a product’s aroma, but can also inhibit microbes. Meanwhile, organic acids produced during fermentation lower a product’s pH, acting as preservatives and flavor enhancers. In essence, these compounds can improve the shelf-life, safety, and sensory qualities of foods while also providing health benefits. This dual functionality is highly attractive for researchers aiming to produce cleaner food products (e.g., those containing fewer synthetic additives) and aligns with consumer demand for natural health-promoting ingredients.

Recent research highlights alternative, underutilized and novel sources of food or components/isolates that can be used to create new, safe and functional products. These include agri-food byproducts, microalgae, seaweed, insect-derived food, fungi, medicinal plants, etc. [13,14,15,16,17,18]. These sources provide unique bioactive profiles and promote food sector sustainability. Extraction methods including ultrasound-assisted, supercritical CO_2_, and microwave-assisted extraction are preferred for their efficiency and sustainability [19]; however, with the chemical diversity of bioactive compounds, advanced analytical strategies have become crucial for identifying and characterizing them. Structural characterization and activity screening through in vitro, in vivo, and in silico methods are key to validating functional properties. Additionally, scientists are actively investigating new processing techniques with a less negative impact on nutritive properties and valuable food constituents. Best practices include the following:

- Using whole natural sources that are high in bioactives (e.g., adding berry purees rich in anthocyanins and flavonols to beverages).
- Food supplementation and fortified foods (addition of isolated bioactive compounds or concentrated/dried extracts to food products to boost their health value).
- Applying measures to stabilize the bioactive compounds.
- Assessing the bioavailability of the compounds, as well as their controlled release in the body.
- Selecting specific protective strains or probiotic cultures to boost the content of certain vitamins (e.g., LAB that synthesize B-vitamins), release bound phenolics from plant fibers, or generate unique bioactive peptides.
- Exploring the synergy of combined bioactives, such as pairing probiotics with prebiotic fibers (synbiotics) or blending different plant extracts to target various health concerns through a single product, developing edible coatings and films infused with natural antimicrobials (like chitosan films with plant extracts), combining bioactives with synergistic nutrients, maintaining clean-label formulations, and ensuring regulatory compliance are essential to successful functional food development.

In addition, these natural, valuable and bioactive ingredients often provide antioxidant, anti-inflammatory, cardioprotective, neuroprotective, and gut-modulatory effects that are essential for promoting overall health and wellness [12,19]. They also play a role in reducing oxidative stress, enhancing metabolic function, supporting immunity, and potentially preventing chronic diseases. Postbiotics and polyphenol-rich diets have shown promise in improving gut and cognitive health, while fermented and fortified foods contribute to overall wellbeing [12,19,20]. It should be noted that bioactives often work synergistically, and that the combination of compounds available in a whole food may offer greater benefits than a single isolated compound.

Despite significant progress, there remain several challenges and frontiers in the field of food bioactive compounds. These include enhancing the bioavailability of the neutral compounds; clarifying precise molecular mechanisms for many effects; expanding the search for new bioactive sources in underutilized plants, marine organisms, and agro-industrial byproducts; ensuring the safety of concentrations of bioactives added to foods, as well as recommended intake levels; and bridging the gap between scientific advancements and consumer perspective and acceptance of the novel solutions. Research should focus on personalized nutrition, sustainable sourcing, and effective communication of health claims to maximize public health impact. The synergy between food science, biotechnology, and nutrition continues to shape the next generation of smarter functional foods. Such foods will not only nourish but also provide targeted benefits (from heart health to cognitive support), helping consumers take charge of their health through their diets.

The primary aim of this Special Issue (SI), “Bioactive Compounds in Foods: New and Novel Sources, Characterization, Strategies, and Applications”, is to investigate both current and emerging sources of valuable natural bioactive compounds, and best practices for incorporating them into food products. The SI comprises ten original research papers and one review; together, these works address recent advances and current knowledge in the proposed field (please see the list of contributions for further details). We believe that further investigations in this scientific field will offer solutions to current global problems and meet the nutritional needs of future generations in a sustainable manner, protecting the planet’s ecosystems.

Finally, we would like to thank all the authors and the reviewers who contributed their work, knowledge and suggestions to this SI, as well as MDPI and the *Foods* journal for their help and support.

## List of Contributions

1. Costa, A.S.G.; Peixoto, J.A.B.; Machado, S.; Espírito Santo, L.; Soares, T.F.; Andrade, N.; Azevedo, R.; Almeida, A.; Costa, H.S.; Oliveira, M.B.P.P.; et al. Coffee Pulp from Azores: A Novel Phytochemical-Rich Food with Potential Anti-Diabetic Properties. *Foods* **2025**, *14*, 306. https://doi.org/10.3390/foods14020306.
2. Nikolić, V.; Žilić, S.; Simić, M.; Šavikin, K.; Stević, T.; Živković, J.; Sarić, B.; Milovanović, D.; Kandić Raftery, V. Characterization and Potential Food Applications of Oat Flour and Husks from Differently Colored Genotypes as Novel Nutritional Sources of Bioactive Compounds. *Foods* **2024**, *13*, 3853. https://doi.org/10.3390/foods13233853.
3. Coyago-Cruz, E.; Barrigas, A.; Guachamin, A.; Heredia-Moya, J.; Zuñiga-Miranda, J.; Vera, E. Bioactive Composition of Tropical Flowers and Their Antioxidant and Antimicrobial Properties. *Foods* **2024**, *13*, 3766. https://doi.org/10.3390/foods13233766.
4. Katsuki, T.; Ogi, K.; Kinno, A.; Kasamatsu, S.; Ihara, H.; Sumitani, H. Inhibition of Amyloid β Accumulation by Protease-Digested Whitebait (Shirasu) in a Murine Model of Alzheimer’s Disease. *Foods* **2024**, *13*, 2858. https://doi.org/10.3390/foods13182858.
5. Murthy, H.N.; Yadav, G.G.; Joseph, K.S.; HS, S.K.; Magi, S.M.; Dewir, Y.H.; Mendler-Drienyovszki, N. Nutritional Value, Fatty Acid and Phytochemical Composition, and Antioxidant Properties of Mysore Fig (*Ficus drupacea* Thunb.) Fruits. *Foods* **2024**, *13*, 2845. https://doi.org/10.3390/foods13172845.
6. Higuchi, J.; Kurogochi, M.; Yamaguchi, T.; Fujio, N.; Mitsuduka, S.; Ishida, Y.; Fukudome, H.; Nonoyama, N.; Gota, M.; Mizuno, M.; et al. Qualitative and Quantitative Analyses of Sialyl O-Glycans in Milk-Derived Sialylglycopeptide Concentrate. *Foods* **2024**, *13*, 2792. https://doi.org/10.3390/foods13172792.
7. Bebek Markovinović, A.; Bosiljkov, T.; Janči, T.; Kostić, M.; Dedović, N.; Lučić, E.; Bavrka, K.; Pavlić, B.; Bursać Kovačević, D. Characterization of Antioxidant Bioactive Compounds and Rheological, Color and Sensory Properties in 3D-Printed Fruit Snacks. *Foods* **2024**, *13*, 1623. https://doi.org/10.3390/foods13111623.
8. Lemoine, C.; Rodrigues, M.J.; Dauvergne, X.; Cérantola, S.; Custódio, L.; Magné, C. A Characterization of Biological Activities and Bioactive Phenolics from the Non-Volatile Fraction of the Edible and Medicinal Halophyte Sea Fennel (*Crithmum maritimum* L.). *Foods* **2024**, *13*, 1294. https://doi.org/10.3390/foods13091294.
9. Politeo, O.; Ćurlin, P.; Brzović, P.; Auzende, K.; Magné, C.; Generalić Mekinić, I. Volatiles from French and Croatian Sea Fennel Ecotypes: Chemical Profiles and the Antioxidant, Antimicrobial and Antiageing Activity of Essential Oils and Hydrolates. *Foods* **2024**, *13*, 695. https://doi.org/10.3390/foods13050695.
10. Cardinali, F.; Belleggia, L.; Reale, A.; Cirlini, M.; Boscaino, F.; Di Renzo, T.; Del Vecchio, L.; Cavalca, N.; Milanović, V.; Garofalo, C.; et al. Exploitation of Black Olive (*Olea europaea* L. cv. Piantone di Mogliano) Pomace for the Production of High-Value Bread. *Foods* **2024**, *13*, 460. https://doi.org/10.3390/foods13030460.
11. Sayas-Barberá, E.; Paredes, C.; Salgado-Ramos, M.; Pallarés, N.; Ferrer, E.; Navarro-Rodríguez de Vera, C.; Pérez-Álvarez, J.Á. Approaches to Enhance Sugar Content in Foods: Is the Date Palm Fruit a Natural Alternative to Sweeteners? *Foods* **2024**, *13*, 129. https://doi.org/10.3390/foods13010129.
